# After more than a decade of soil moisture deficit, tropical rainforest trees maintain photosynthetic capacity, despite increased leaf respiration

**DOI:** 10.1111/gcb.13035

**Published:** 2015-09-22

**Authors:** Lucy Rowland, Raquel L. Lobo‐do‐Vale, Bradley O. Christoffersen, Eliane A. Melém, Bart Kruijt, Steel S. Vasconcelos, Tomas Domingues, Oliver J. Binks, Alex A. R. Oliveira, Daniel Metcalfe, Antonio C. L. da Costa, Maurizio Mencuccini, Patrick Meir

**Affiliations:** ^1^ School of GeoSciencesUniversity of EdinburghEdinburghUK; ^2^ Forest Research CentreSchool of AgricultureUniversity of LisbonLisbonPortugal; ^3^ Earth and Environmental SciencesLos Alamos National LaboratoryLos AlamosCAUSA; ^4^ EMBRAPA Amazônia OrientalBelémBrasil; ^5^ AlterraWageningen URWageningenthe Netherlands; ^6^ Departamento de BiologiaFFCLRP ‐ Universidade de São PauloRibeirão PretoBrasil; ^7^ Centro de GeosciênciasUniversidade Federal do ParáBelémBrasil; ^8^ Department of Physical Geography and Ecosystem ScienceLund UniversityLundSweden; ^9^ ICREA at CREAF08193 Cerdanyola del VallésBarcelonaSpain; ^10^ Research School of BiologyAustralian National UniversityCanberraAustralia

**Keywords:** drought, leaf dark respiration, photosynthetic capacity, through‐fall exclusion, tropical rainforest

## Abstract

Determining climate change feedbacks from tropical rainforests requires an understanding of how carbon gain through photosynthesis and loss through respiration will be altered. One of the key changes that tropical rainforests may experience under future climate change scenarios is reduced soil moisture availability. In this study we examine if and how both leaf photosynthesis and leaf dark respiration acclimate following more than 12 years of experimental soil moisture deficit, via a through‐fall exclusion experiment (TFE) in an eastern Amazonian rainforest. We find that experimentally drought‐stressed trees and taxa maintain the same maximum leaf photosynthetic capacity as trees in corresponding control forest, independent of their susceptibility to drought‐induced mortality. We hypothesize that photosynthetic capacity is maintained across all treatments and taxa to take advantage of short‐lived periods of high moisture availability, when stomatal conductance (*g*_s_) and photosynthesis can increase rapidly, potentially compensating for reduced assimilate supply at other times. Average leaf dark respiration (*R*_d_) was elevated in the TFE‐treated forest trees relative to the control by 28.2 ± 2.8% (mean ± one standard error). This mean *R*_d_ value was dominated by a 48.5 ± 3.6% increase in the *R*_d_ of drought‐sensitive taxa, and likely reflects the need for additional metabolic support required for stress‐related repair, and hydraulic or osmotic maintenance processes. Following soil moisture deficit that is maintained for several years, our data suggest that changes in respiration drive greater shifts in the canopy carbon balance, than changes in photosynthetic capacity.

## Introduction

Tropical rainforests are the world's most productive and biodiverse terrestrial ecosystem (Beer *et al*., 2010), harbouring a substantial store of carbon, particularly in Amazonia because of its area (Grace *et al*., 2014). How long tropical rainforests will remain a store of carbon remains uncertain, and may depend upon the intensity and/or frequency of future drought events (Phillips *et al*., 2009; Gatti *et al*., 2014). Small shifts in photosynthetic and autotrophic respiratory process responses to seasonal reductions in moisture have previously been shown to shift the carbon balance of tropical rainforests (Bonal *et al*., 2008; Meir *et al*., 2008; Rowland *et al*., 2014); however, how photosynthesis and respiration interact and acclimate to multi‐year drought in tropical rainforest remains poorly understood.

Evidence from studies of drought‐induced changes in photosynthesis and leaf respiration suggest that the effects on photosynthesis are greater than on autotrophic respiration (Ciais *et al*., 2005; Flexas *et al*., 2006; Meir *et al*., 2008; Catoni & Gratani, 2014; Chastain *et al*., 2014; Doughty *et al*., 2015). Drought‐induced reductions in leaf‐level photosynthesis have been found to occur through either reductions in stomatal conductance (*g*_s_) and/or mesophyll conductance (Miranda *et al*., 2005; Flexas *et al*., 2006; Stahl *et al*., 2013; Catoni & Gratani, 2014; Chastain *et al*., 2014), or reductions in biochemical efficiency (Flexas & Medrano, 2002; Lawlor & Cornic, 2002; Reddy *et al*., 2004; Galmes *et al*., 2013). During short‐term or less severe drought events, down‐regulation of metabolic processes as a result of low *g*_s_ is thought to be the main limitation on photosynthesis (Flexas & Medrano, 2002; Flexas *et al*., 2006).

Photosynthetic capacity represents the maximum rate at which leaves can photosynthesize in optimum conditions. Provided optimum conditions are realized reductions in photosynthetic capacity will restrict total photosynthesis; in turn increased photosynthetic capacity will only increase photosynthesis in the absence of other limitations such as stomatal conductance or light. If a canopy photosynthesis is optimized to maximize light‐use efficiency (Hirose *et al*., 1988; Kull & Kruijt, 1999), photosynthetic capacity is likely to be maintained, or potentially increased during these less severe drought events to maximize photosynthetic output at a lower *g*_s_. However, as the severity and length of drought increases, accumulation of reactive oxygen species (ROS) can occur if *g*_s_ drops below a threshold value; this will result in more permanent reductions in photosynthesis through biochemical changes causing cellular damage (Flexas *et al*., 2006). Cellular damage occurring during severe drought stress can reduce leaf metabolic function by: decreasing ribulose1, 5‐biphosphate (RuBP) regeneration capacity, ATP synthesis or ATP content in the leaf, and Rubisco activity; or, by increasing leaf photochemical reaction rates (chlorophyll fluorescence) or photoinhibition (Flexas & Medrano, 2002; Reddy *et al*., 2004; Flexas *et al*., 2006; Galmes *et al*., 2013). Consequently, under severe drought‐stressed conditions photosynthetic capacity [the maximum rates of carboxylation of Rubisco (*V*_cmax_) and/or electron transport (*J*_max_)] may decline, driving changes in total leaf‐scale photosynthesis.

The processes involved in leaf respiration remain less well understood than for photosynthesis (Atkin & Macherel, 2009; Atkin *et al*., 2013, 2015); particularly in tropical rainforests and in relation to drought (Meir *et al*., 2008; Meir & Woodward, 2010; Rowland *et al*., 2014). Short‐term, acute water stress is generally found to inhibit *R*_d_ because of either reduced substrate supply from photosynthesis with respiratory substrates being diverted to form compounds for osmotic regulation, or through direct reductions in mitochondrial respiratory capacity (Atkin & Macherel, 2009; Ayub *et al*., 2011; Catoni & Gratani, 2014; Chastain *et al*., 2014; O'brien *et al*., 2015). However, it is also possible that *R*_d_ can increase during the periods of drought if substrate demand for processes such as hydraulic maintenance and repair (Brodersen & Mcelrone, 2013), and phloem transport regulation (Mencuccini & Hölttä, 2010) increases, or if drought conditions lead to a greater need to oxidize ROS or other redox equivalents, elevating photorespiration (Atkin & Macherel, 2009). Evidence of increasing *R*_d_ during drought is limited but has been reported for both crop plants and forests (Miranda *et al*., 2005; Atkin & Macherel, 2009; Varone & Gratani, 2015).

The responses to long‐term drought stress are likely to differ from those of short‐term drought because certain processes, which may be sustainable over short time periods, are unlikely to facilitate long‐term drought survival. Over longer time periods structural or physiological acclimation may occur to facilitate adaptation to drought conditions at a whole tree scale. Acclimation to drought within trees has been reported to occur across various time‐scales from seasonal to multi‐annual. In response to seasonal drought trees have been reported to increase their leaf‐scale water use efficiency (WUE: unit photosynthesis per unit water loss; Bonal *et al*., 2007), reduce the rate at which *R*_d_ increases with temperature to minimize leaf carbon loss (Crous *et al*., 2011) as well as to increase leaf mass per area (Sperlich *et al*., 2015). Over multi‐year drought conditions the key acclimation processes may differ as whole tree‐scale structural changes become possible and perhaps necessary to maintain a positive carbon balance. These structural changes may include altered root growth patterns (Metcalfe *et al*., 2007), changes in canopy leaf area (Brando *et al*., 2008; Metcalfe *et al*., 2010a) or changes in carbon allocation and stem growth (Brando *et al*., 2008; Da Costa *et al*., 2010; Metcalfe *et al*., 2010b). Such structural changes are likely to compensate for, or facilitate further, any changes in physiology. Increased leaf‐level respiration was found after 5 years of experimental drought, on a mix of shaded and sunlit leaves of a tropical rainforest (Metcalfe *et al*., 2010b). Maintaining elevated respiration would require enhanced substrate use, thus also requiring acclimation responses in either allocation to growth or from carbon storage. However, whether elevated *R*_d_ is related to acclimation responses in photosynthetic capacity or WUE, or even if elevated *R*_d_ can be maintained during long‐term soil moisture deficit (e.g. for more than a decade), has yet to be determined.

In tropical rainforest, where species diversity is high and drought sensitivity has been shown to vary with taxonomic identity (Nepstad *et al*., 2007; Da Costa *et al*., 2010), there is likely to be a range of photosynthetic and respiratory acclimation responses to drought in any forest stand (Stahl *et al*., 2013). Understanding these processes and their possible acclimation in the context of long‐term drought in tropical rainforests is key to constraining the variability which currently exists in model predictions of such processes in future climate change scenarios (Galbraith *et al*., 2010; Huntingford *et al*., 2013; Powell *et al*., 2013; Rowland *et al*., 2015), or in more conceptual models (Meir *et al*., 2015a).

We present wet and dry season measurements of photosynthetic capacity (*V*_cmax_ and *J*_max_), *R*_d_, *g*_s_, photosynthesis in saturating light and ambient atmospheric CO_2_ concentration (*A*_sat_), WUE and leaf nutrient content for tropical rainforest which has experienced 13 years of experimental soil moisture deficit imposed by a through‐fall exclusion (TFE) treatment and corresponding, adjacent control forest. We also compare variables for tree taxa that have been previously determined as sensitive or resistant to drought in terms of their mortality response to experimental soil moisture deficit (Da Costa *et al*., 2010). The results are compared with earlier measurements of photosynthesis and respiration made during the first 3 years of the experiment. Using these data we test the following hypotheses: (1) *V*_cmax,_ *J*_max_ and *R*_d_, change following prolonged soil moisture deficit; (2) Drought sensitivity (specified as the drought‐induced mortality risk of specific tree genera) constrains the physiological response (*V*_cmax_, *J*_max_, *R*_d_, *A*_sat_ and WUE) of a tree to seasonal or experimental drought and (3) the relationships between *R*_d_, photosynthetic capacity and nutrient content change following prolonged soil moisture deficit.

## Materials and methods

### Site

This study was performed at a long‐term TFE experiment in a tropical rainforest, located in the Caxiuanã National Forest Reserve in the eastern Amazon (1°43′S. 51°27W), on *terra firme* forest, with yellow oxisol soils (Ruivo & Cunha, 2003). The site is 15 m above sea level, has a mean annual rainfall between 2000 and 2500 mm and a pronounced dry season between June and November when monthly rainfall is <100 mm.

The experiment comprises two 1 ha plots, a treatment (TFE) and a control. In the TFE plot plastic panels and gutters are supported at a height of 1–2 m and exclude 50% of the incident rainfall (Da Costa *et al*., 2010). A corresponding control plot is sited <50 m from the TFE, where there has been no manipulation of incident through‐fall. As with many large‐scale ecosystem manipulations, this experiment was prohibitively large and expensive to permit replication. The potential insights derived from long‐term monitoring at large‐scale were favoured over the use of smaller plots replicated over large geographical areas (Carpenter, 1996; Osmond *et al*., 2004). In addition, the large horizontal extent of many tree roots in this forest ecosystem made a small‐plot design inappropriate. As described in earlier publications from this experiment (Fisher *et al*., 2007; Meir *et al*., 2009, 2015b; Da Costa *et al*., 2010), and as also performed in other unreplicated large‐scale experiments such as at Hubbard Brook, pretreatment data were obtained before the start of the experiment, enabling comparison over time as well as by treatment (see also Nepstad *et al*., 2002; Davidson *et al*., 2004). Perimeter trenching to 1–2 m depth was used to avoid lateral in‐flow of soil water, and the litter on each panel was transferred manually to the forest floor immediately below it every few days to maintain these biogeochemical inputs to the soil. For more experimental details see (Fisher *et al*., 2007; Meir *et al*., 2009; Da Costa *et al*., 2010). The TFE experiment has been maintained continuously since January 2002 until the present (2015), and thus, prior to this study, the trees had experienced a 50% reduction in through‐fall for 12–13 years.

### Species selection and measurement protocol

#### Campaigns during 2013–2014

In October–November 2013 (peak dry season) and July 2014 (peak wet season) leaf photosynthesis and leaf dark respiration measurements were performed on 21 canopy‐top trees from the control and TFE forest (42 samples in total; Table 1). The selected trees were from six genera, with 3–6 replicates per genus. We tested the criteria used by Da Costa *et al*. (2010; See Table S1) to identify how sensitive these genera were to drought stress. We found strong consistency with the Da Costa *et al*. (2010) results using six additional years of data, from (i.e. from 2009 to 2014). Of the six genera employed, three were classed as sensitive, and three as resistant to drought in terms of their drought‐induced mortality response (Table S1). Branches from the canopy‐tops of 21 trees from the TFE plot and 20 trees from the control plot were cut between 10:00 and 15:00 h, over several days in each sampling season. The stems were then immediately immersed in water, and recut to reconstitute the water supply to leaves, as also performed elsewhere for the chosen gas exchange measurements (Domingues *et al*., 2010). After recutting underwater, branches were left to stabilize in a canopy opening, in full ambient irradiance for a minimum of 30 min before leaf measurements were conducted. Gas exchange measurements were performed using three LICOR 6400 portable photosynthesis systems (LI‐COR, Lincoln, NE, USA), which were regularly cross‐calibrated through checking repeated measures on the same leaves to ensure consistency among measurements. Following careful field tests on‐site, no significant effect of branch cutting was found on our measurements (See measurement protocol below, and Fig. S1).

**Table 1:** Genus, species with genus and sensitivity to drought‐induced mortality (see Table S1) for the number of trees sampled in the control and TFE plots for the 2013 and 2014 analysis of *R*_d_, *V*_cmax_, *J*_max_, *A*_sat_, *g*_s_, leaf nutrients and LMA

Genus	Species sampled within Genus	Vulnerability	No° sampled on control plot	No° sampled on TFE plot
*Eschweilera*	*Coriacea, grandiflora, pedicellata*	Vulnerable	5	6
*Licania*	*Membranacea, octandra*	Resistant	3	3
*Manilkara*	*Bidentata*	Vulnerable	3	3
*Pouteria*	*Anomala*	Vulnerable	3	3
*Protium*	*Tenuifolium, paniculatum*	Resistant	3	3
*Swartzia*	*Racemosa*	Resistant	3	3

The *V*_cmax_ and *J*_max_ were derived by measuring photosynthesis at different concentrations of CO_2_ (50–2000 ppm) in the leaf chamber. The *A*–C_i_ curves (*C*_i_, the CO_2_ concentration of the leaf intercellular spaces) were performed using a saturating photosynthetically active radiation (PAR) intensity of 2000 μmol m^−2^ s^−1^, determined from light curves performed separately on each genus. The *V*_cmax_ and *J*_max_ were calculated from the *A*–C_i_ curves after temperature‐correction to 25 °C using the equations from Sharkey *et al*. (2007). Model fitting was carried out using the optim function in R, which optimizes the fit of an *A*–*C*_i_ curve to the data (R.3.0.2; R Core Team). As we were unable to measure mesophyll conductance we assumed it to be infinite (i.e. there to be no mesophyll conductance limitation) in our fitting procedure, rather than fitting this parameter separately, to avoid introducing unknown biases in our comparisons of control with treatment. Saturating photosynthesis (*A*_sat_) was measured separately to the *A*–*C*_i_ curves at a CO_2_ concentration of 400 ppm and a PAR intensity of 2000 μmol m^−2^ s^−1^. The *A*_sat_ was corrected to 25 °C using standard temperature constants from Sharkey *et al*. (2007). To standardize for variations in *A*_sat_ caused by differences in the internal CO_2_ concentration (*C*_i_) when leaves are exposed to the same external CO_2_ concentrations *A*_sat_ was corrected to a common *C*i value; the median *C*_i_ of all *A*_sat_ measurements was used as the common *C*_i_ value. The *A*_sat_ was only measured if photosynthesis and *g*_s_ had remained stable within the LI‐COR chamber for a minimum period of 10 min. Intrinsic leaf water use efficiency (WUE) was calculated as *A*_sat_ divided by the *g*_s_ measured at *A*_sat._ As the branches were cut, measured WUE may not be identical to WUE of intact branches at the top of the canopy; however, the effects of cutting on photosynthesis and maximum *g*_s_ were nonsignificant (Fig. S1), and sampling among tree taxa was balanced between the control and TFE (Table 1), thereby minimizing potential biases due to branch cutting. Leaf dark respiration (*R*_d_) was measured after keeping the leaf in complete darkness inside tinfoil, for 30 min. The *R*_d_ measurements were corrected to 25 °*C* using Equation 1, where *R*_dt_ is respiration at the leaf temperature inside the leaf chamber, *T* is the leaf temperature inside the leaf chamber and the *Q*_10_ factor 2.2 represents the factor by which respiration increases for a 10 °C increase in leaf temperature (Atkin & Tjoelker, 2003).

$$R_{d}=R_{\mathrm{dt}}\cdot 2.2^{\left(\frac{25-T}{10}\right)}$$

#### Measurement campaigns during 2001–2003

The *A*–*C*_i_ curves were also recorded on attached leaves from trees adjacent to two walk‐up observation towers in each plot, in both the wet and dry seasons of 2001 (pre‐TFE treatment), 2002 and 2003. Samples were taken from 11 trees of 10 different species in the control, and nine trees of nine different species in the TFE; the total number of samples taken per period are shown in Table S2. As some of the tree canopies were partially shaded on each plot (Table S2), saturating PAR intensity for these curves was set at 1000 μmol m^−2^ s^−1^. To ensure comparability between the 2001–2003 and the 2013–2014 protocols, *A*–*C*_i_ curves and leaf respiration were remeasured on these same trees in 2013 and 2014 (Table S2). However, only one *A*–*C*_i_ curve and two *R*_d_ measurements could be determined in the TFE because of tree mortality and loss of the branches close to the tower since 2003. The *V*_cmax_ and *J*_max_ were calculated from the *A*–*C*_i_ data using the same method as above. During the 2001–2003 campaigns, light response curves were also performed on the same leaves employed for *A*–*C*_i_ curves in both control and TFE plots. Previously published data on *R*_d_ at Caxiuanã (Metcalfe *et al*., 2010a) were derived from these light response curves.

#### Additional measurement campaigns

Additional *R*_d_ data were obtained in 2007 on sunlit and shaded leaves from cut branches obtained from both the control and TFE plots (Metcalfe *et al*., 2010a; Table S3). All these values of *R*_d_ were corrected to 25 °C using Equation 1. The same trees sampled in 2007 by Metcalfe *et al*. (2010a) were resampled where possible in this study, in peak dry season 2013, and peak wet season 2014 (Table S3). In summary, we synthesized leaf measurements of *V*_cmax,_ *J*_max_ and *R*_d_ from the experiment over the full experimental period, 2001–2014, to test the hypotheses laid out above.

### Nutrient and leaf mass analysis

All leaves used for gas exchange were collected, scanned to obtain leaf area, dried to constant mass and weighed. Leaf area was measured from the scanned images using the image j software (http://imagej.nih.gov/ij/), which, together with the dry mass of the leaf was used to calculate leaf mass per area (LMA, g m^−2^), and its reciprocal, specific leaf area (SLA m^−2 ^kg^−1^). Following removal of the petiole and the main vein, and grinding of the dry tissue, nitrogen (*N*) and phosphorus (*P*) content of all leaves were measured in the EMBRAPA laboratories in Belem, Para, Brazil. The *N* concentrations of 0.1 g subsamples were determined by dry combustion using a LECO CNS‐2000 analyser (LECO Corporation, Michigan) and tested against laboratory standards. The *P* concentrations of 0.1 g subsamples were determined by the molybdate method (Murphy & Riley, 1962) after digestion using sulphuric acid and peroxide.

### Statistics

All statistical analyses were done in r (Version 3.02, R Core Team). Mixed effects modelling was performed following standard methods (Zuur *et al*., 2009), using the lm4 package. Models were formulated to test for a drought treatment effect on *V*_cmax_, *J*_max_, *R*_d_, *N*,* P,* LMA and SLA where plot, sensitivity status (whether a tree belonged to a taxon which was considered sensitive or resistant to drought‐induced mortality based on the data from this experiment; Table S1) and season were considered as fixed effects, and individual tree, nested within genus was considered as a random effect. Models were also formulated to test if a single relationship was maintained between *V*_cmax_, *J*_max_, *R*_d_ and leaf nutrients plus the combination of SLA (or LMA), in both the control and TFE, independent of drought stress. In these models *N*,* P* and either LMA or SLA were considered fixed effects and individual tree, nested within genus, was considered as a random effect. All tests were performed on both a leaf area and a leaf mass basis. To find the most appropriate model for a variable, a likelihood ratio test with one degree of freedom was used to compare models with and without the variable of interest. Models were tested both with and without variable interactions. All other tests for significance throughout the study were done using the Wilcoxon rank‐sum test statistic, and significant results were considered as those with a *P* value <0.05.

## Results

### Campaigns during 2013–2014

**Figure 1:**
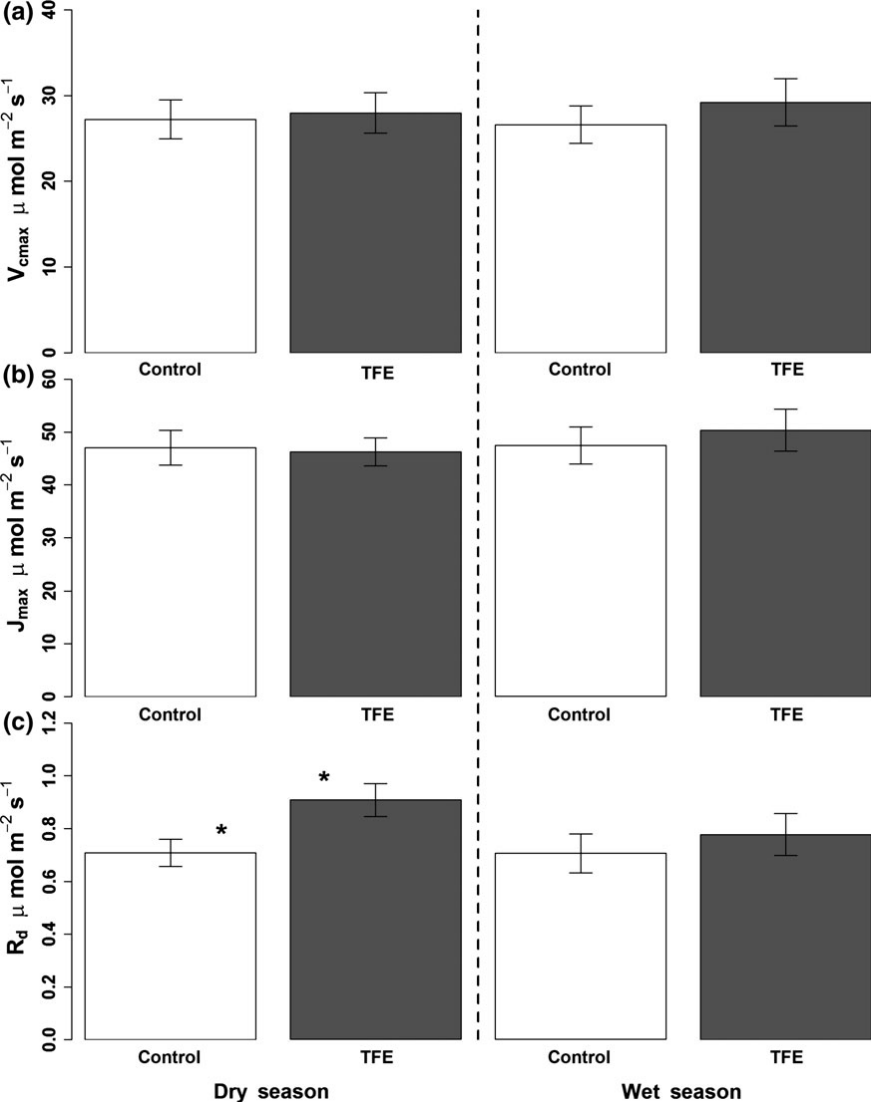
Average *V*_cmax_ (a), *J*max (b) and *R*_d_ (c) expressed in μmol m^−2^ s^−1^ in the control (C; white) and TFE (grey) plots, in peak dry season 2013 (November) and peak wet season 2014 (June). Error bars show the standard error.

**Figure 2:**
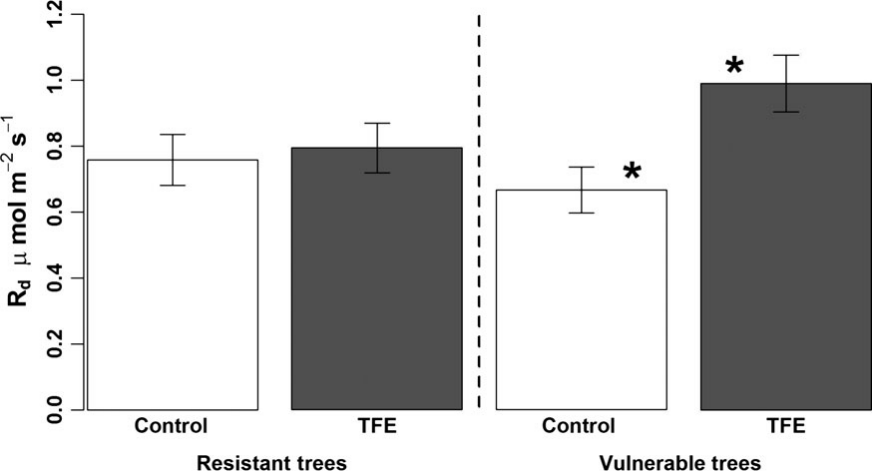
*R*_d_ for the resistant and vulnerable tree taxa in the control (white) and TFE (grey) plot in peak dry season of 2013. Columns with a * indicates significant difference with *P* < 0.05. Error bars show standard error.

**Figure 3:**
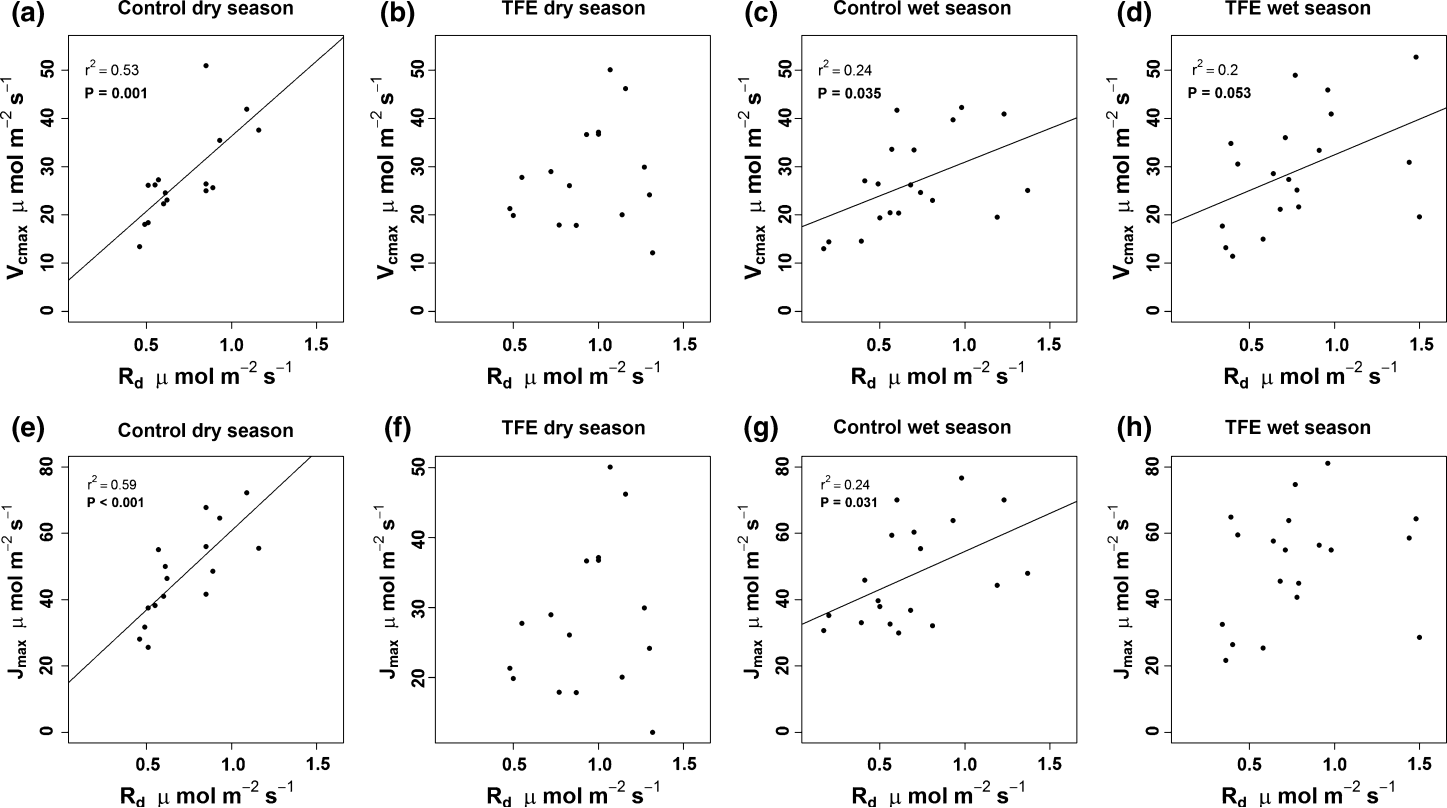
Relationships between *V*_cmax_ and *R*_d_ (a–d) and *J*_max_ and *R*_d_ (e–h) during the wet and dry season in the control and TFE plot. If the linear line is significant (*P* < 0.05), the linear relationship, correlation coefficient (*r* ^2^) and significance value (*P*) are shown.

No significant differences were found for *V*_cmax_ and *J*_max_ between the control and the TFE plots, in either wet or dry seasons (Fig. 1a & b). The average values for *V*_cmax_ and *J*_max_ across both seasons were 29.1 ± 1.8 μmol m^−2^ s^−1^ (one standard error) and 51.1 ± 2.8 μmol m^−2^ s^−1^, respectively in the control plot, and 26.8 ± 1.6 μmol m^−2^ s^−1^ and 45.2 ± 1.9 μmol m^−2^ s^−1^ respectively, in the TFE. The average *J*_max_: *V*_cmax_ ratio was 1.8 ± 0.1 in both the control and the TFE plot. Leaf respiration (*R*_d_) was significantly elevated (*P* = 0.03) in the TFE (0.9 ± 0.1 μmol CO_2_ m^−2^ s^−1^) relative to the control plot (0.7 ± 0.1 μmol CO_2_ m^−2^ s^−1^) during the dry season, but no significant differences existed in the wet season (Fig. 1c). These results were very similar when expressed on a leaf mass basis (Fig. S2). The significant increase in *R*_d_ in the TFE during the dry season was driven by elevated respiration in the tree taxa vulnerable to drought‐induced mortality, rather than by an overall increase across all trees and taxa (Fig. 2; Table S1). *R*_d_ in drought‐vulnerable taxa increased 48.5 ± 3.6% (*P* = 0.02; Fig. 2) in the TFE, relative to the average *R*_d_ of the same taxa in control plot; in contrast no significant changes in the respiration rates of the drought‐resistant taxa were observed. The relationships of *R*_d_ with *V*_cmax_ and *J*_max_ were stronger in the control plot than in the TFE (Fig. 3), particularly during the dry season when correlation coefficients were >0.5 in the control plot while no significant relationships were found for the TFE (Fig. 3).

**Figure 4:**
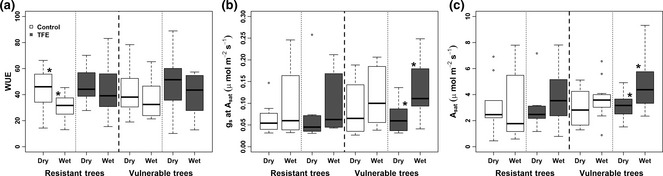
Box plots of water use efficiency (WUE; a), stomatal conductance (*g*_s_; b) and light‐saturated photosynthesis at 400 ppm of CO_2_ (*A*_sat_) during peak dry season 2013 and peak wet season 2014 for vulnerable and resistant tree taxa in the control (white) and TFE (grey) plots. * indicates significant difference with *P *<* *0.05.

Tree vulnerability status seemed to influence seasonal patterns of WUE, *A*_sat_ and *g*_s_. Resistant taxa in the control plot experienced significantly increased WUE in the dry season (*P* = 0.03; Fig. 4a) by delivering an elevated *A*_sat_ at a similar *g*_s_ to the wet season. This did not occur in either the vulnerable or resistant trees in the TFE because *A*_sat_ was not elevated and *g*_s_ was reduced in the dry season (Fig. 4b, c). The vulnerable trees in the TFE experienced a significant 34.0 ± 5.1% (*P* = 0.02) reduction in *A*_sat_ from the peak of the wet season to the peak of the dry season, which was related to a drop of 22.9 ± 9.6% in peak *g*_s_ (*P* = 0.02; Fig. 4b, c).

The mixed effect model analysis demonstrated that none of the treatment effect (plot), season, vulnerability status or any combination of these variables contributed significantly to predicting *V*_cmax_, *J*_max_, *N* and *P*, on either a mass or area basis, and the same was also true for modelling LMA and SLA (Table 2). As no plot or seasonal difference was detectable in any of these variables, we found that across both plots and both seasons LMA was significant in the modelling of *V*_cmax_ on an area basis and *N* was significant in the modelling of *V*_cmax_ and *J*_max_ on a mass basis (Table 2). In contrast the interaction of plot, vulnerability status and season was a highly significant component of the best model of *R*_d_, on both a mass and area basis (Table 2). The importance of plot, season and drought sensitivity in explaining the variance in *R*_d_ prevented a more general model of *R*_d_ based on *N*,* P*, LMA or SLA, or any combination of these variables, from being significant (Table 2).

**Table 2:** Mixed effect model results on a leaf area and a leaf mass basis, to test for: (a) the drought treatment effect on *V*_cmax_, *J*_max,_ *R*_d_, nitrogen (N), phosphorus (P), leaf mass per area (LMA), and specific leaf area (SLA), using the treatment effect [control vs. TFE plot (Pl)], tree vulnerable or resistant status (V), and season (S) as fixed variables and tree nested within genus as the random component of the model. (b) the effect of nutrients and leaf area and mass on *V*_cmax_, *J*_max_ and *R*_d_, using N, P and either LMA or SLA as fixed variables and tree nested within genus as the random component of the model. The significance of the intercept (*P*) and valid fixed variables are shown as well as the proportion of the model variance accounted for by the random component of the model (*R* variance, %). * indicates variable interaction

Models to test treatment effects			
Y variable	Fixed variables tested	Significant fixed variables	*R* Variance, %
(a)			
*V*_cmax_ area basis	Pl, V, S	None	64.41
*J*_max_ area basis	Pl, V, S	None	43.53
*R*_d_ area basis	Pl, V, S	Pl*V*S (*P *=* *0.02)	31.54
N area basis	Pl, V, S	None	78.34
P area basis	Pl, V, S	None	17.36
LMA	Pl, V, S	None	52.46
*V*_cmax_ mass basis	Pl, V, S	None	52.35
*J*_max_ mass basis	Pl, V, S	None	32.61
*R*_d_ mass basis	Pl, V, S	Pl*V*S (*P *=* *0.02)	43.60
N mass basis	Pl, V, S	None	71.21
P mass basis	Pl, V, S	None	30.78
SLA	Pl, V, S	None	60.30
(b)			
*V*_cmax_ area basis	N, P, LMA	LMA (*P *<* *0.01)	68.86
*J*_max_ area basis	N, P, LMA	None	45.01
*R*_d_ area basis	N, P, LMA	None	4.62
*V*_cmax_ mass basis	N, P, SLA	N (*P *<* *0.01)	44.98
*J*_max_ mass basis	N, P, SLA	N (*P *<* *0.01)	14.97
*R*_d_ mass basis	N, P, SLA	None	27.73

### Comparison between the 2013–2014 results and earlier campaigns

**Figure 5:**
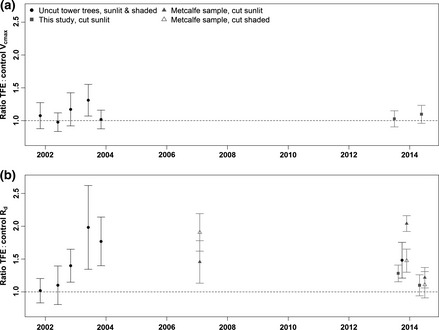
Ratio of TFE: control plot *V*_cmax_ (a) and *R*_d_ (b) values for various measurement campaigns made at the Caxiuanã TFE experiment from prior to the start of the experiment (2001) to 2014; see Methods for details. The symbols indicate the differences in the sample selection. ‘Uncut tower trees’ are data from leaves on attached branches accessed via a walk‐up through‐canopy tower. ‘Metcalfe sample’ are from leaves on the same trees sampled by Metcalfe *et al*. (2010a). This study used cut branches with fully sunlit leaves and the results from Fig. 2 are replotted here.

Over the 13 years of the TFE experiment, the ratio of TFE: control *V*_cmax_ remained close to 1.0, suggesting leaf photosynthetic capacity was not altered throughout the experiment (Fig. 5a). In contrast, measurements of TFE: control *R*_d_ over 13 years of drought were significantly ≤ 1.0. This conclusion was robust to tests carried out to determine the potential influence of using leaf samples taken in different seasons from different species using fundamentally different measurement methods (i.e. cut and uncut branches from shaded and sunlit canopy positions). The magnitude of the difference in *R*_d_ between the control and TFE plots is likely to have been affected by the composition of tree taxa, where sampling of taxa differed, and canopy height in the samples, and also the seasonal conditions, but despite these sources of potential variation, the overall *R*_d_ signal was significant and remained elevated in the TFE plot (Fig. 5b).

## Discussion

Although the TFE does not simulate the atmospheric effects of drought, it allows us to isolate the impacts of prolonged soil moisture deficit on leaf physiology, a process which is poorly simulated in ecosystem models, yet responsible for substantial uncertainty in modelled climate change predictions for Amazonia (Powell *et al*., 2013; Rowland *et al*., 2015). Following 13 years of soil moisture deficit in the TFE there has been no change in the photosynthetic capacity of the trees, but a 48.5 ± 3.6% increase in the leaf‐level *R*_d_ of drought‐vulnerable trees in the TFE relative to the control (Fig. 1 & 5, Table 2). Our measured values of both *V*_cmax_ and *J*_max_ lie well within the ranges previously measured in tropical rainforests (Carswell *et al*., 2000; Coste *et al*., 2005; Domingues *et al*., 2005; Meir *et al*., 2007; Van De Weg *et al*., 2012) and we demonstrate that substantial long‐term reductions in soil moisture availability in the TFE (L. Rowland, A.C.L. da Costa, M. Mencuccini, D.R. Galbraith, R.S. Oliveira, O.J. Binks, A.A.R. Oliveira, A.M. Pullen, C.E. Doughty, D.B. Metcalfe, S.S. Vasconcelos, L.V. Ferreira, Y. Malhi, J. Grace & P. Meir, unpublished results ) have no impact on the values of these photosynthetic parameters. In addition, no significant difference was observed in *A*_sat_ between the control and TFE plots in either vulnerable or resistant trees (Fig. 4c); however, seasonal differences in *A*_sat_ and *g*_s_ at *A*_sat_ were greater for the drought‐vulnerable trees in the TFE (Fig. 4b, c). These results suggest that, in the TFE, even the taxa which are vulnerable to drought‐induced mortality can achieve the same maximum photosynthetic rates as the control trees. However, as photosynthetic capacity does not equate to realized photosynthesis, it is possible that the TFE trees may still suffer from lower photosynthesis for example caused by lower daily average g_s_.

Interestingly the taxa which are resistant to drought‐induced mortality in the TFE showed no obvious signs of acclimation of photosynthetic capacity, *R*_d_ or *g*_s_ at *A*_sat_ and this may suggest that these trees acclimate to the drought conditions in other ways, such as reducing individual canopy leaf area or by increasing root water uptake (Metcalfe *et al*., 2007, 2010a,b). However, maintenance of photosynthetic capacity in the droughted leaves may instead be an acclimation response itself; provided sufficient light and nutrients are available, investment in maintaining *V*_cmax_ and *J*_max_, at the expense of other investments within the tree, would compensate for canopy‐scale reductions in *g*_s_ and *C*_i_, allowing maximization of photosynthetic output during wetter periods when *g*_s_ is elevated. This would, in theory, optimize light‐use efficiency in the canopy (Hirose *et al*., 1988; Meir *et al*., 2002; Niinemets *et al*., 2015), provided no other long‐term drought‐related stress was occurring within the leaf. This potential for the droughted trees to maintain the same maximum photosynthetic output is consistent with the recent demonstration at this TFE experiment of no depletion of stored leaf, stem and branch nonstructural carbohydrates (NSC), and no reduced investment into carbon‐demanding processes such as growth following 13 years of experimental drought (L. Rowland, A.C.L. da Costa, M. Mencuccini, D.R. Galbraith, R.S. Oliveira, O.J. Binks, A.A.R. Oliveira, A.M. Pullen, C.E. Doughty, D.B. Metcalfe, S.S. Vasconcelos, L.V. Ferreira, Y. Malhi, J. Grace & P. Meir, unpublished results ).

Previously, Metcalfe *et al*. (2010a) found *R*_d_ to be elevated in the TFE but their sample was taken from a subset of mostly shaded leaves, from trees of widely differing species in the control and TFE, without replication by species or mortality‐risk. Using a sample design that balances tree taxa between plots across vulnerability status we show here that the increased *R*_d_ is a long‐term acclimation response in the TFE, and is attributable to the higher respiration of certain taxa vulnerable to drought‐induced mortality, during the driest parts of the year. Increased *R*_d_ in response to low soil moisture has been observed elsewhere in Amazonia (Miranda *et al*., 2005) and has also been reported in Mediterranean species, as, albeit during shorter term drought conditions, elevations in *R*_d_ were associated with low resistance to water stress in three taxa that had higher plant maintenance costs during drought (Varone & Gratani, 2015). It is therefore possible that an enhanced respiration cost during drought is a trait which has not been selected against in the Amazon because of its adaptive advantage. Significant reductions in *g*_s_ and *A*_sat_ in the drought‐vulnerable trees suggest they are more hydraulically vulnerable during the dry season (Fig. 4). Consequently, elevated *R*_d_ in these taxa may be explained by an increased necessity for hydraulic repair (Brodersen & Mcelrone, 2013), for processing of ROS (Ribas‐Carbo *et al*., 2005; Atkin & Macherel, 2009) and/or an increase in photorespiration (Atkin & Macherel, 2009), or, counter‐intuitively here, use of increased substrate supply, which may occur if processes involved in carbon use or transport are more impaired by drought than photosynthesis (Körner, 2013). We, thus, hypothesize that the increased carbon use (this study) and high mortality (Da Costa *et al*., 2010; Table S1) in vulnerable trees in the TFE plot (Fig. 2) are caused by a reduced ability to cope with the physiological stress caused by prolonged reductions in soil water content, but that this physiological stress is not severe enough to decrease photosynthetic capacity.

The biochemistry of photosynthesis and *R*_d_ are related, such that a short‐term down‐regulation of photosynthesis has frequently been observed to lead to a down‐regulation of *R*_d_ (Flexas *et al*., 2006; Catoni & Gratani, 2014; Chastain *et al*., 2014). Reductions in the ratio of photosynthesis to *R*_d_ during drought have been reported elsewhere in the literature (Miranda *et al*., 2005; Atkin & Macherel, 2009; Catoni & Gratani, 2014; Chastain *et al*., 2014); we find this result at the Caxiuanã TFE experiment, but unlike the cited studies we find this to be driven only by increased *R*_d_ rather than decreased photosynthetic capacity. Our results also demonstrate that the relationship between photosynthetic capacity (*V*_cmax_ and *J*_max_) and *R*_d_ are much weaker in the TFE than in control forest (Fig. 3), and that the standard relationships expected between *R*_d_ and *N*,* P*, LMA or SLA (Atkin *et al*., 2015) are not found because of a significant treatment effect on *R*_d_, but not on *N*,* P*, LMA or SLA (Table 2). This may suggest that *N* and *P* do not substantially limit *R*_d_, in these plots. These results also suggest that the normally tight coupling of leaf respiratory processes with photosynthetic processes, and their relationships with leaf nutrient content and LMA or SLA are disrupted during long‐term drought stress in tropical trees, and new model formulations are needed to simulate this response to drought.

This study examines how photosynthetic capacity and leaf dark respiration (*R*_d_) acclimate to long‐term (>12 years) soil moisture deficit in a tropical rainforest. Using a sampling design with a balanced selection of tree taxa between control and treatment we demonstrate that, following 12–13 years of soil moisture deficit, photosynthetic capacity is unchanged even in taxa which are prone to drought‐induced mortality. The rate of *R*_d_ in the dry season in the experimentally droughted forest was, however, significantly elevated because of a 48.5 ± 3.6% increase in the *R*_d_ of taxa which are prone to drought‐induced mortality. We conclude that, even after a severe and prolonged reduction in soil moisture availability, trees are able to maintain the same photosynthetic capacity, despite increasing *R*_d_. Such results suggest that better models of acclimation in *R*_d_ and overall gas exchange may be necessary to accurately simulate the responses of tropical rainforests to drought.

## Supporting information


**FigureS1.** *V*_cmax_ (a, μmol m^−2^ s^−1^) and *J*_max_ (b, μmol m^−2^ s^−1^) and maximum *g*_s_ (c, *g*_smax_; μmol m^−2^ s^−1^) for A–*C*_i_ curves performed on eight uncut *in‐situ* fully sunlit leaves and repeated on the same leaves following the branch being cut and re‐cut underwater (see Methods section). No significant differences were found between uncut (white) and cut (grey) values for either *V*_cmax_, *J*_max_, or *g*_smax_. Sample selection included reachable fully sunlit trees from the towers in the TFE (two branches) and control plot (two branches), and four fully sunlit tree canopies from outside of the plots.

**FigureS2.** Repeat of Fig. 1 on a mass basis showing average *V*_cmax_ (a), *J*_max_ (b) and *R*_d_ (c) measured in μmol g^−1^ s^−1^ in the control (C; white) and TFE (grey) plot in peak dry season 2013 (November) and peak wet season 2014 (June). Error bar shows the standard error.

**Table S1.** The number of living trees in 2001, the number of trees dead from 2001 to 2014 and the percentage mortality of the common genera (>10 individuals per plot) in the control and TFE plot since the start of the experiment (2002).
**Table S2.** Trees sampled for *A*–*C*_i_ curves (indicated with x) and for leaf respiration (grey shading) from the towers in the control and TFE plot from 2001 to 2014.
**Table S3.** Numbers of trees sampled from around the towers in the control and TFE plot, for respiration measurements made originally by Metcalfe *et al*. (2007) and repeat samples taken in this study in dry season 2013 and wet season 2014.
