# Supplementary figures and images for: After more than a decade of soil moisture deficit, tropical rainforest trees maintain photosynthetic capacity, despite increased leaf respiration
Source: Glob Chang Biol. 2015 Sep 22;21(12):4662–72. doi: 10.1111/gcb.13035 (PMC4989466; doi:10.1111/gcb.13035)

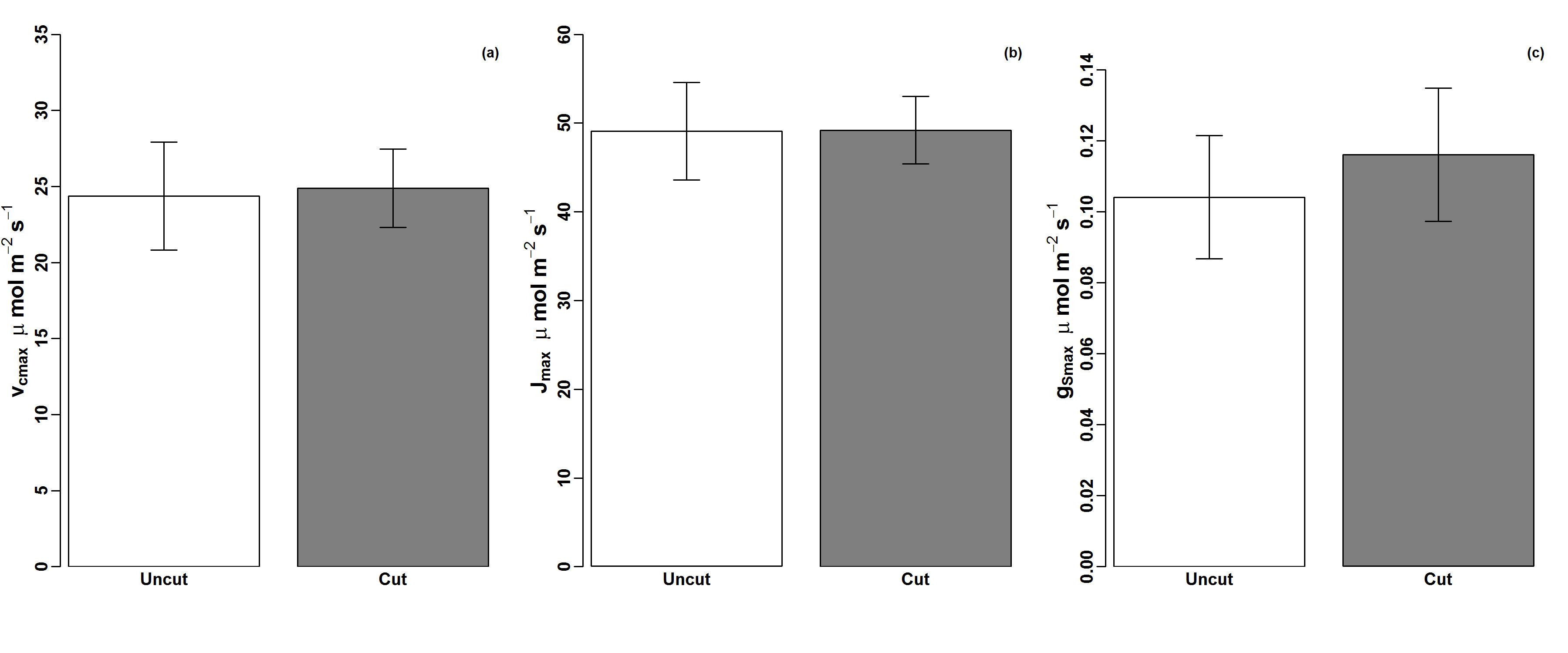

Supplement: Supplementary file 1 — FigureS1. V cmax (a, μmol m−2 s−1) and J max (b, μmol m−2 s−1) and maximum g s (c, g smax; μmol m−2 s−1) for A–C i curves performed on eight uncut in‐situ fully sunlit leaves and repeated on the same leaves following the branch being cut and re‐cut underwater (see Methods section). No significant differences were found between uncut (white) and cut (grey) values for either V cmax, J max, or g smax. Sample selection included reachable fully sunlit trees from the towers in the TFE (two branches) and control plot (two branches), and four fully sunlit tree canopies from outside of the plots. [file GCB-21-4662-s001.jpeg]

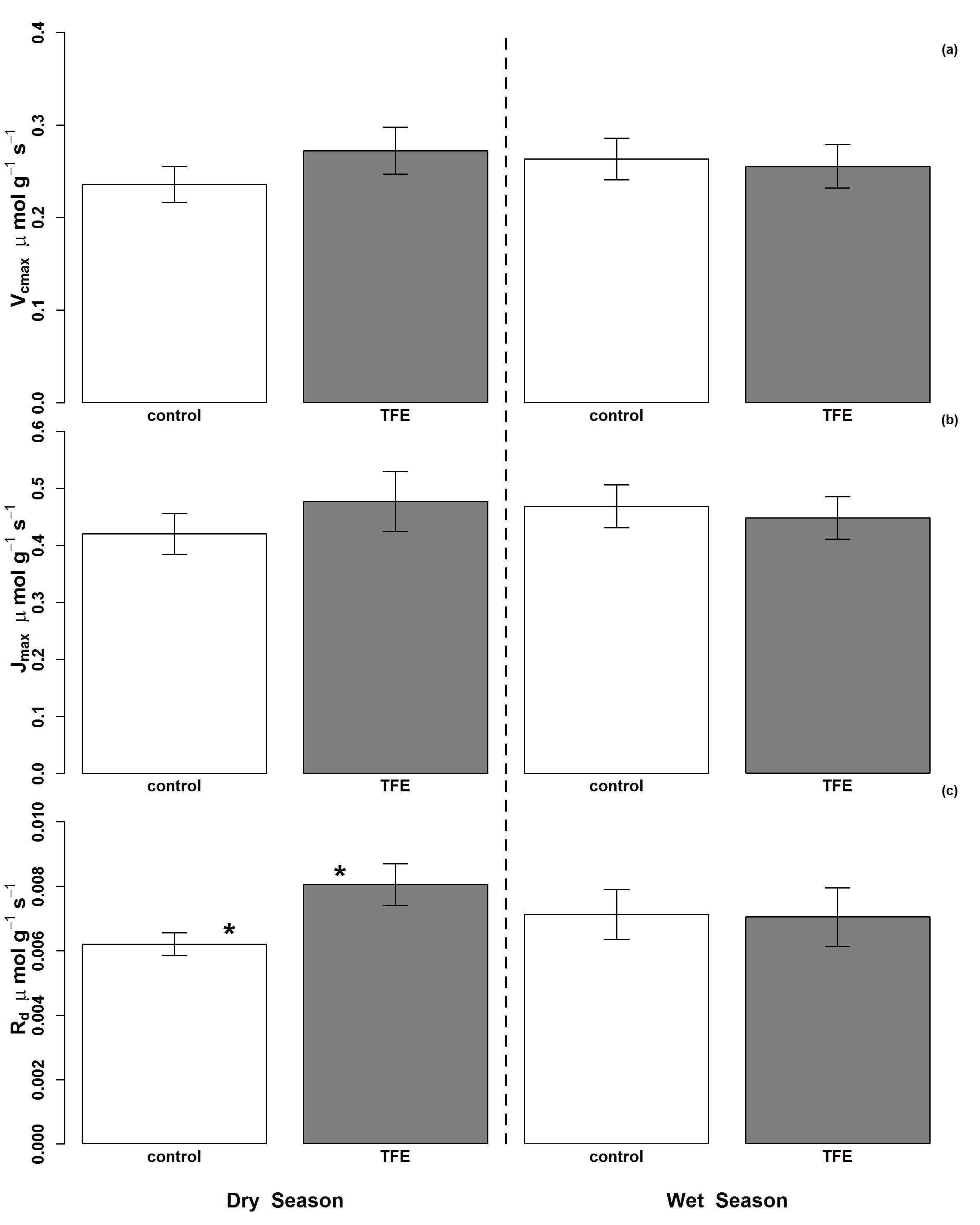

Supplement: Supplementary file 2 — FigureS2. Repeat of Fig. 1 on a mass basis showing average V cmax (a), J max (b) and R d (c) measured in μmol g−1 s−1 in the control (C; white) and TFE (grey) plot in peak dry season 2013 (November) and peak wet season 2014 (June). Error bar shows the standard error. [file GCB-21-4662-s002.jpeg]
