# Supplementary material for: After more than a decade of soil moisture deficit, tropical rainforest trees maintain photosynthetic capacity, despite increased leaf respiration
Source: Glob Chang Biol. 2015 Sep 22;21(12):4662–72. doi: 10.1111/gcb.13035 (PMC4989466; doi:10.1111/gcb.13035)
Supplement: Supplementary file 3 — Table S1. The number of living trees in 2001, the number of trees dead from 2001 to 2014 and the percentage mortality of the common genera (>10 individuals per plot) in the control and TFE plot since the start of the experiment (2002). Table S2. Trees sampled for A–C i curves (indicated with x) and for leaf respiration (grey shading) from the towers in the control and TFE plot from 2001 to 2014. Table S3. Numbers of trees sampled from around the towers in the control and TFE plot, for respiration measurements made originally by Metcalfe et al. (2007) and repeat samples taken in this study in dry season 2013 and wet season 2014. [file GCB-21-4662-s003.docx]

**Supplementary Tables**

Table S1: The number of living trees in 2001, the number of trees dead from 2001–2014 and the percentage mortality of the common genera (>10 individuals per plot) in the control and TFE plot since the start of the experiment (2002). The difference between the number of dead trees per genus in the control and TFE plot is also shown. Highlighted and bold rows show trees which are considered to be sensitive to drought based on the criteria from da Costa et al. (2010): a mortality rate in the TFE plot which is >1.5 times that of the control, provided at least two individuals more died in the TFE.

|  | **Control Plot** | | | **TFE Plot** | | |  |
| --- | --- | --- | --- | --- | --- | --- | --- |
| **Genus** | **No ° alive 2001** | **No° dead 2014** | **% mortality** | **No ° alive 2001** | **No ° dead 2014** | **% mortality** | **Difference in No° dead trees** |
| **Trees with ≥10 individuals per plot** | | | | | | | |
| ***Pouteria*** | **44** | **4** | **9.09** | **32** | **6** | **18.75** | **2** |
| *Licania* | 29 | 4 | 13.79 | 15 | 2 | 13.33 | -2 |
| *Vouacapoua* | 17 | 2 | 11.76 | 4 | 0 | 00.00 | -2 |
| *Protium* | 16 | 4 | 25.00 | 15 | 5 | 33.33 | 1 |
| *Swartzia* | 16 | 4 | 25.00 | 9 | 1 | 11.11 | -3 |
| ***Eschweilera*** | **13** | **1** | **7.69** | **19** | **4** | **21.05** | **3** |
| ***Manilkara*** | **12** | **0** | **0.00** | **14** | **3** | **21.42** | **3** |
| ***Inga*** | **10** | **2** | **20.00** | **12** | **4** | **33.33** | **2** |
| **Trees with ≤10 individuals in the control but ≥10 individuals in the TFE** | | | | | | | |
| ***Lecythis*** | **3** | **0** | **0.00** | **17** | **4** | **23.52** | **4** |
| *Tetragastris* | 4 | 2 | 50.00 | 14 | 3 | 21.4 | 1 |
| *Minquartia* | 8 | 2 | 25.00 | 9 | 2 | 22.22 | 0 |
| ***Micropholis*** | **7** | **1** | **14.29** | **10** | **5** | **50.00** | **3** |
| ***Stachyarrhena*** | **4** | **1** | **25.00** | **8** | **3** | **37.50** | **2** |

Table S2: Trees sampled for *A*–*C*_i_ curves (indicated with x) and for leaf respiration (grey shading) from the towers in the control and TFE plot from 2001–2014.

| **Tree** | **Species** | **Canopy Height (m)** | **Canopy Shading** | **2001 Dry** | **2002 Wet** | **2002 Dry** | **2003 Wet** | **2003 Dry** | **2003 Wet** | **2014 Wet** |
| --- | --- | --- | --- | --- | --- | --- | --- | --- | --- | --- |
| Control 1 | *Quiina florida* | 2 | Shaded | x |  | x | x | x |  |  |
| Control 2 | *Pouteria lateriflora* | 2 | Shaded | x | x | x | x | x |  |  |
| Control 3 | *Protium heptaphyllum* | 4 | Shaded |  | x | x | x | x |  |  |
| Control 4 | *Pouteria* | 4 | Shaded |  | x | x | x | x |  |  |
| Control 5 | *Quiina florida* | 10 | Shaded |  | x | x | x | x |  |  |
| Control 6 | *Duguetia echinophora* | 18 | Semi sunlit | x | x |  | x | x | x | x |
| Control 7 | *Hasseltia floribunda* | 10 | Semi sunlit | x |  |  |  | x |  |  |
| Control 8 | *Mezilaurus mahuba* | 10 | sunlit | x | x | x | x |  | x | x |
| Control 9 | *Licania heteromorpha* | 18 | sunlit | x | x | x | x | x | x | x |
| Control 10 | *Manilkara bidentata* | 30 | Sunlit |  | x |  | x | x | x | x |
| Control 11 | *Manilkara bidentata* | 30 | Sulnit |  | x | x |  |  | x | x |
| TFE 1 | *Licania canescens* | 12 | Semi sunlit | x | x | x | x | x |  |  |
| TFE 2 | *Duguetia echinophora* | 14 | Shaded |  | x | x | x | x |  |  |
| TFE 3 | *Hirtela bicornis* | 14 | Semi sunlit | x |  |  |  | x | x | x |
| TFE 4 | *Mouriri duckeana* | 16 | Semi sunlit | x | x | x | x | x |  |  |
| TFE 5 | *Licaria armeniaca* | 18 | Sunlit |  | x |  | x |  |  |  |
| TFE 6 | *Hirtela bicornis* | 22 | Sunlit | x | x | x | x | x |  |  |
| TFE 7 | *Lecythis confertiflora* | 28 | Sunlit |  |  | x |  | x |  |  |
| TFE 8 | *Manilkara paraensis* | 30 | Sunlit |  | x | x | x |  |  |  |
| TFE 9 | *Swartzia racemosa* | 30 | Sunlit | x | x | x | x |  |  |  |

Table S3: Numbers of trees sampled from around the towers in the control and TFE plot, for respiration measurements made originally by Metcalfe *et al.,* (2007) and repeat samples taken in this study in dry season 2013 and wet season 2014. Samples are taken from a range of species which were originally reachable by a pruning pole to cut branches from around the towers; where possible we replicated this sample, however, some tree species remained unidentified in Metcalfe *et al.,* (2007).

|  | **2007 (Dry Season; from Metcalfe et al., 2007)** | | **2013 (Dry Season; repeat sample, this study)** | | **2014 (Wet Season; repeat sample, this study)** | |
| --- | --- | --- | --- | --- | --- | --- |
| **Plot** | **Control** | **TFE** | **Control** | **TFE** | **Control** | **TFE** |
| **Top canopy (fully sunlit samples, from >15 m height)** | 20 | 9 | 12 | 3 | 8 | 10 |
| **Lower canopy (all or partially shaded leaves from >15 m height)** | 13 | 19 | 11 | 4 | 9 | 11 |
